# Tinea Corporis Masquerading as a Diffuse Gyrate Erythema: Case Report and a Review of Annular Lesions Mimicking a Dermatophyte Skin Infection

**DOI:** 10.7759/cureus.8935

**Published:** 2020-06-30

**Authors:** Darlene Diep, Antoanella Calame, Philip R Cohen

**Affiliations:** 1 Medicine, Burrell College of Osteopathic Medicine, Las Cruces, USA; 2 Dermatology/Dermatopathology, Compass Dermatopathology, San Diego, USA; 3 Dermatology, Scripps Memorial Hospital, La Jolla, USA; 4 Dermatology, San Diego Family Dermatology, National City, USA

**Keywords:** corporis, dermatitis, dermatophyte, diffuse, erythema, gyrate, hyphae, incognito, masquerade, tinea

## Abstract

Tinea is a superficial fungal infection of the skin. Gyrate erythemas are reactive conditions that present as annular red lesions. A 61-year-old woman was diagnosed with tinea corporis whose skin lesions morphologically mimicked a gyrate erythema. She presented with diffuse annular plaques affecting the left side of her chest and abdomen that did not respond to a combination antifungal-corticosteroid cream for six-month duration. The appearance and clinical differential diagnosis included a gyrate erythema. Initial evaluation of the skin biopsy from the lesion’s edge demonstrated a spongiotic dermatitis, and staining for fungal organisms was negative. However, deeper sections and a different fungal stain revealed hyphae in the stratum corneum and established a diagnosis of tinea corporis. The PubMed database was used to review the following terms: tinea corporis, gyrate erythema, and tinea incognito. Relevant papers and references cited in those papers that were generated by the search were used. Tinea corporis, especially if previously treated with topical corticosteroids, can masquerade as other dermatoses including a gyrate erythema. Correlation of clinical presentation and pathology findings is essential, especially if the biopsy results do not confirm the suspected clinical diagnosis. Consideration to perform deeper sections or additional special stains or both should also be entertained when the initial pathology observations do not support the presumptive diagnosis based on clinical morphology and history.

## Introduction

Gyrate erythema includes annular lesions with erythematous raise borders. Scale may be present or absent. They can be solitary or multiple, fixed, or expanding [1]. 

Tinea corporis is a dermatophyte infection of the skin. It is typically asymptomatic. It usually presents as a solitary red plaque with a scaly border [2]. 

A woman with a chronic dermatophyte infection that morphologically mimicked a diffuse gyrate erythema is described. Initial evaluation of her skin biopsy only reveals a spongiotic dermatitis that was consistent with a diagnosis of a gyrate erythema. However, deeper sections and additional special staining to detect fungal organisms established the diagnosis of tinea corporis. 

## Case presentation

A 61-year-old Nepalese woman, Fitzpatrick skin type IV (light brown skin that burns minimally and tans easily), presented for evaluation of a generalized, diffuse skin rash of six-month duration. The lesions not only itched, but also were painful and had a burning sensation; her symptoms worsened with sweating. 

The rash had begun as a single lesion that continued to enlarge. Subsequently, new lesions also appeared on her chest and axilla. The patient’s primary care physician prescribed a topical cream that consisted of an antifungal (clotrimazole 1%) and a high-potency corticosteroid (betamethasone dipropionate 0.05%). The cream was applied twice a day for several months without improvement; the skin lesions continued to increase in size and number. 

**Figure 1:**
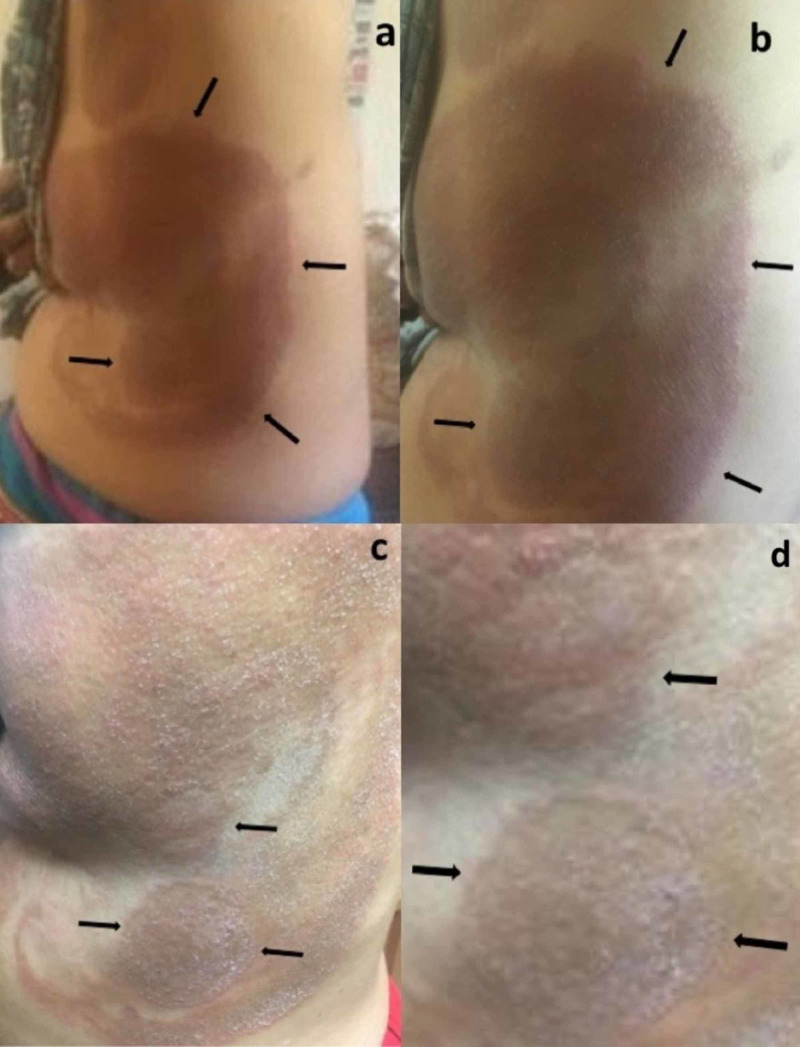
Clinical presentation of tinea corporis mimicking a gyrate erythema Distant (a) and closer (b-d) views of multiple annular lesions that have erythematous scaly borders (black arrows) surrounding macular brown patches in the central area on the left axilla, chest, and abdomen of a 61-year-old woman. The lesions continued to expand while being treated with a topical cream (clotrimazole 1%-betamethasone 0.05%).

She was referred to a dermatologist, who initially evaluated her using telemedicine. Cutaneous examination showed several large patches with raised scaly borders that extended from the left axilla to left chest and left lower abdomen; the lesions also extended from her mid flank to her mid chest and umbilicus. The central portions of the lesions were flat and hyperpigmented, consistent with post-inflammatory hyperpigmentation (Figure 1). She was asked to come into the office to receive a skin biopsy. 

**Figure 2:**
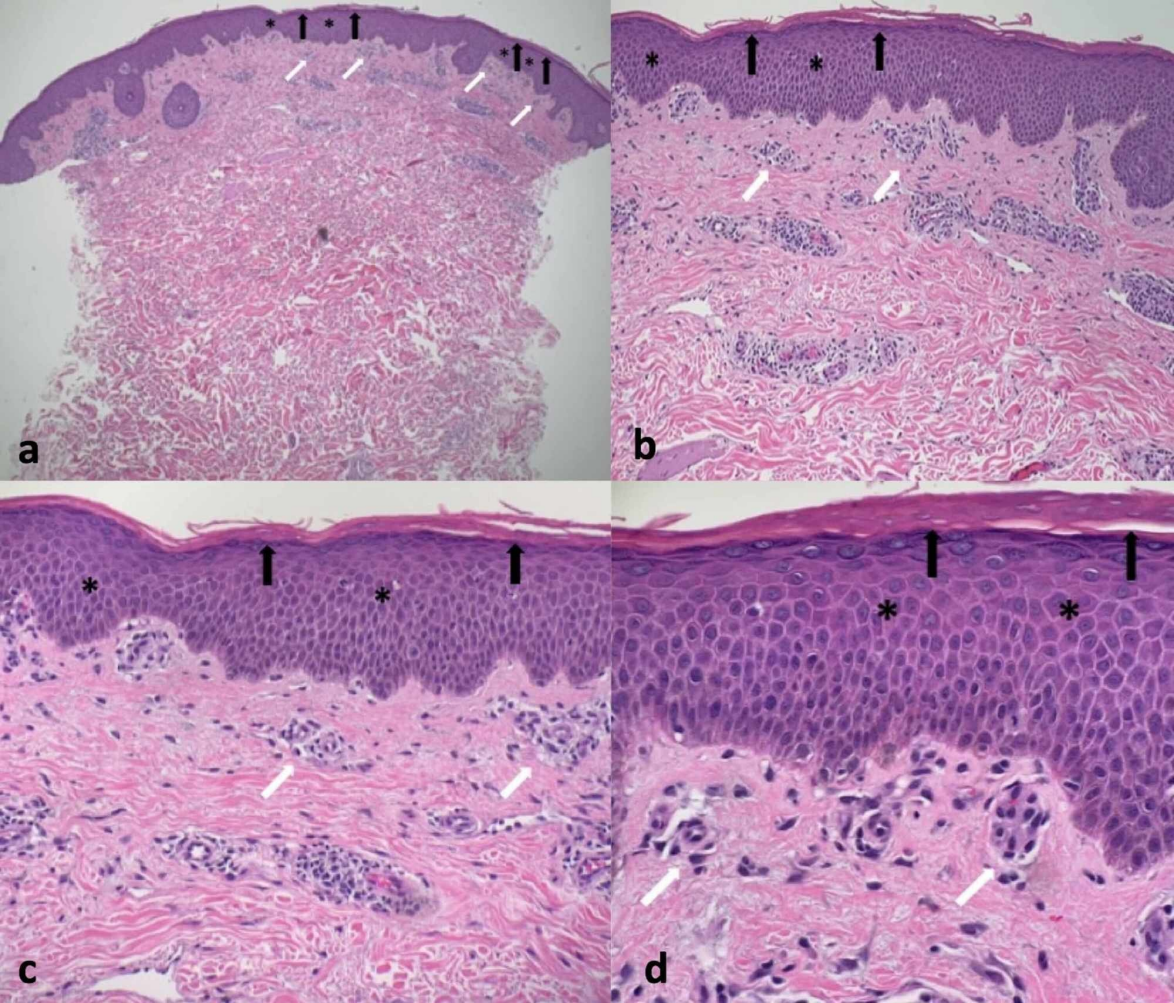
Microscopic presentation of tinea corporis mimicking a gyrate erythema on hematoxylin and eosin stained sections Distant (a) and closer (b-d) views of the hematoxylin and eosin stained sections show compact hyperkeratosis consistent of both orthokeratosis and focal parakeratosis (black arrows); hyphae are not observed in stratum corneum. There is acanthosis (asterisk) and spongiosis of the epidermis. There is a perivascular infiltrate of lymphocytes and edema in the upper dermis (white arrows) (hematoxylin and eosin: a= x4; b= x10, c= x20, d= x40).

A punch biopsy at the edge of her skin lesion was performed. Microscopic examination of the specimen showed hyperkeratosis consisting of both orthokeratosis and focal parakeratosis, acanthosis, and spongiosis. Subepidermal edema and superficial perivascular infiltration of lymphocytes were present in the dermis; there was no evidence of a lymphoproliferative disorder (Figure 2).

**Figure 3:**
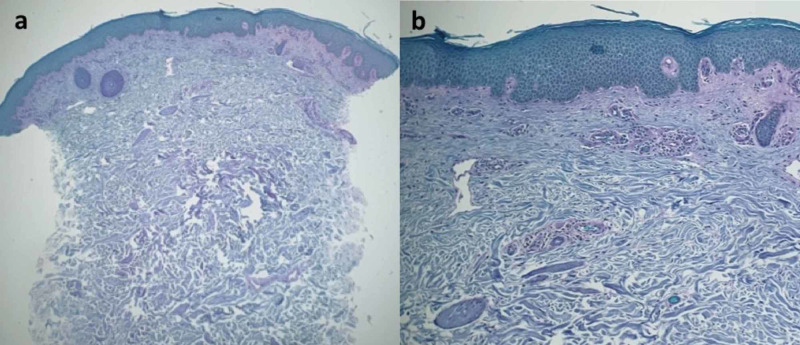
Tinea corporis masquerading as gyrate erythema: microscopic examination of periodic acid-Schiff stained sections Distant (a) and closer (b) views of the tissue specimen after staining with periodic acid-Schiff stain. Fungal organisms are not observed in the stratum corneum (periodic acid-Schiff: a=x4, b= x40).

A special stain, periodic acid-Schiff (PAS) stain, was negative for hyphae (Figure 3); however, hyphae may be rare in tinea corporis patients who have previously been treated with antifungal medication. 

**Figure 4:**
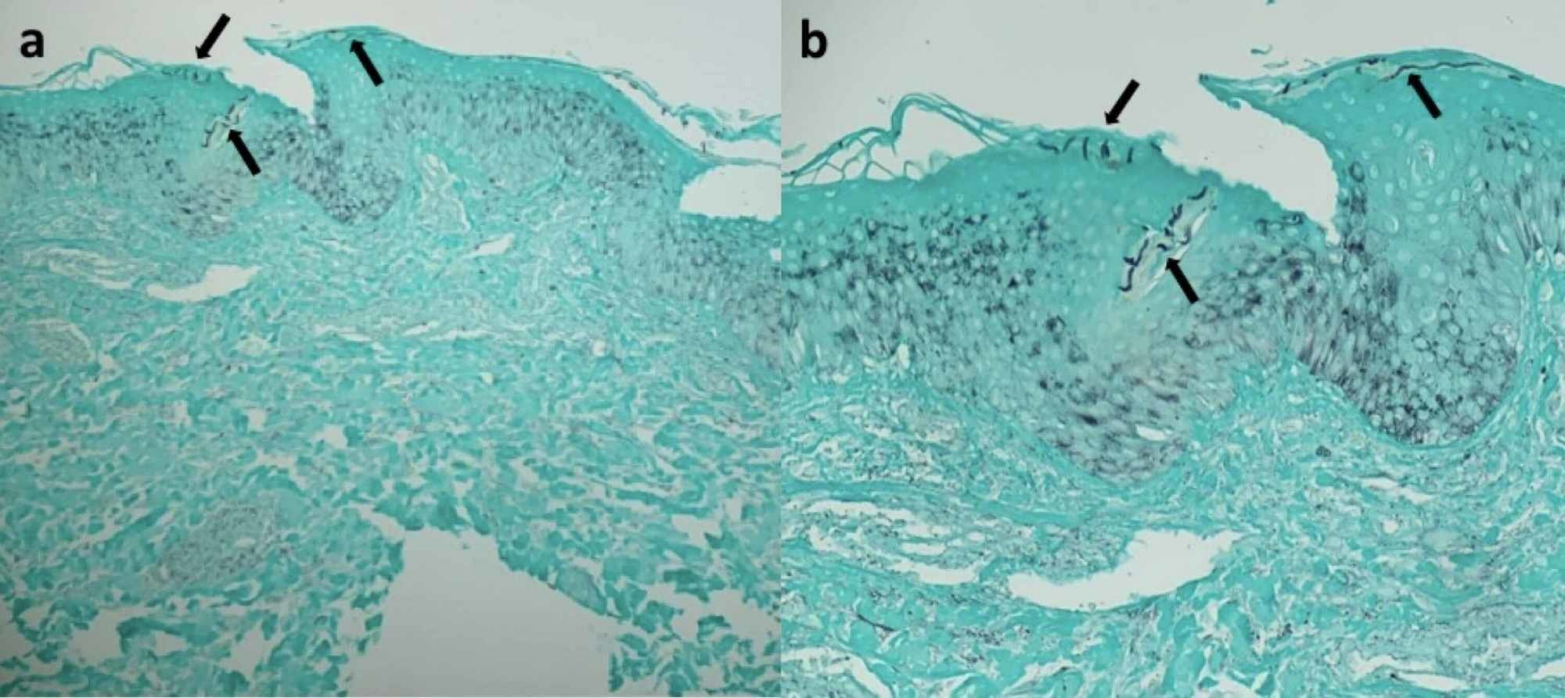
Tinea corporis masquerading as gyrate erythema: microscopic examination of Gomori methenamine silver stained sections Distant (a) and closer (b) views of the tissue specimen after staining with Gomori methenamine silver stain. Numerous fungal hyphae, which appear as black linear organisms are prominent in the stratum corneum (black arrows) (Gomori methenamine silver: a= x20, b= x40).

Although a gyrate erythema (such as erythema annulare centrifugum) and mycosis fungoides (a variant of cutaneous T-cell lymphoma) had originally been considered in the clinical differential diagnosis prior to the biopsy, the possibility of tinea incognito (tinea corporis that has been treated with corticosteroid) was favored. Therefore, the clinician contacted the dermatopathologist to reevaluate the tissue specimen. Deeper sections and an additional special stain to detect fungal organisms, Gomori methenamine silver (GMS) stain, were performed. Numerous hyphae were identified in the stratum corneum of the epidermis (Figure 4). 

Correlation of the clinical history, lesion morphology, and pathology findings established a definitive diagnosis of tinea corporis. The patient was treated with topical (ketoconazole 2% cream twice daily) and systemic (terbinafine 250 mg daily for four weeks) antifungal therapy. Her symptoms resolved, and the lesions stopped enlarging. Follow-up examination, after completing treatment, only showed residual macular brown areas consistent with post-inflammatory hyperpigmentation. 

## Discussion

Gyrate erythemas include erythema annulare centrifugum and erythema gyratum repens. Erythema annulare centrifugum is a nonspecific dermatosis; it can be idiopathic, related to a medication, or associated with an id reaction to a superficial fungal infection [3]. Erythema gyratum repens, and less commonly erythema annulare centrifugum, is a paraneoplastic dermatosis associated with underlying malignancy [3,4]. 

Other annular lesions with central clearing include erythema chronicum migrans and subacute cutaneous lupus erythematosus. Erythema chronicum migrans occurs at the site of the blacklegged tick bite in patients with Lyme disease; it typically presents as an expanding red annular lesion with central clearing [5]. Subacute cutaneous lupus erythematosus can present as either psoriasiform plaques or scaly annular lesions [6]. 

Tinea is a superficial fungal infection of hair, skin, and nails. When tinea involves the scalp, it is referred to as tinea capitis; infection of the nails is referred to as tinea unguium. When fungal infection extends along hair follicles underneath the epidermis, it is referred to as Majocchi’s granuloma. Dermatophyte infection located on the hands and feet is known as tinea manuum and tinea pedis, respectively. Fungal infection of the body is known as tinea corporis, and infection of the groin is referred to as tinea cruris [2]. 

Tinea corporis typically presents as red plaques with scaly borders. It can be a single lesion or there can be multiple lesions. Characteristically, the lesions slowly enlarge. 

The clinical presentation of tinea corporis can be variable (Table 1) [3-14]. It can present as erythema, papules, or plaques. Recently, tinea corporis has also been observed to appear as generalized erythroderma [15].

**Table 1:** Annular lesions in the clinical differential diagnosis of tinea corporis

Morphology	Conditions	References
Gyrate erythema	Erythema annulare centrifugum	[3,4,7]
	Erythema chronicum migrans	[5,7]
	Erythema gyratum repens	[3,7]
Papules	Granuloma annulare	[8]
	Sarcoidosis	[9]
	Secondary syphilis	[10]
Plaques	Mycosis fungoides	[11]
	Nummular dermatitis	[12]
	Pityriasis rosea	[13]
	Psoriasis vulgaris	[14]
	Subacute cutaneous lupus erythematosus	[6]

If tinea corporis is suspected clinically, a potassium hydroxide preparation to evaluate the scaling edge of the lesion can confirm the diagnosis when hyphae are visualized. However, if the tinea corporis lesions have been misinterpreted as dermatitis, the subsequent application of a topical corticosteroid can alter the clinical morphology. Under these circumstances, referred to as tinea incognito, the dermatophyte infection can mimic other conditions, including a gyrate erythema similar to our patient [1,16,17]. 

A biopsy of the dermatophyte-suspected skin lesion can confirm the diagnosis. However, in a hematoxylin and eosin stained section, the features of tinea corporis can be very subtle and can be missed by the pathologist, as in our patient. However, additional levels or staining with special stains or both can establish the diagnosis. In our patient, hyphae were not observed after staining with PAS stain; yet, on deeper sections, they were readily observed after staining with GMS stain. 

In summary, when a dermatophyte skin infection is suspected, the diagnosis can be confirmed by performing a potassium hydroxide preparation of a skin scraping. However, tinea corporis lesions treated with corticosteroids (tinea incognito) may be modified and can mimic other conditions including a gyrate erythema. A biopsy from the lesion’s leading edge, to be evaluated using hematoxylin and eosin and special stains that highlight fungal organisms, can establish the diagnosis. When the diagnosis of a dermatophyte infection is suspected clinically, but not confirmed on pathology, it may be reasonable to pursue reevaluation of the tissue specimen by performing deeper sections and additional special stains for detecting fungal organisms.

## Conclusions

Tinea corporis can masquerade as a gyrate erythema, especially when the lesions have treated with corticosteroids. A woman presented with an enlarging rash that had been treated with an antifungal and corticosteroid cream. The initial stains of the biopsied lesion did not demonstrate hyphae; however, upon evaluation of deeper sections and further special stains to detect fungal organisms, the suspected diagnosis of tinea corporis was confirmed. When the pathology evaluation does not confirm the suspected clinical diagnosis, additional studies of the tissue specimen may establish the diagnosis.
